# Spin Hall Effect of Double-Index Cylindrical Vector Beams in a Tight Focus

**DOI:** 10.3390/mi14020494

**Published:** 2023-02-20

**Authors:** Alexey A. Kovalev, Victor V. Kotlyar

**Affiliations:** 1 Image Processing Systems Institute of the RAS—Branch of FSRC “Crystallography & Photonics” of the RAS, 151 Molodogvardeyskaya St., 443001 Samara, Russia; 2 Samara National Research University, 34 Moskovskoe Shosse, 443086 Samara, Russia

**Keywords:** cylindrical vector beam, double-index cylindrical vector beam, tight focus, Richards-Wolf theory, spin angular momentum, optical spin Hall effect, orbital angular momentum spectrum

## Abstract

We investigate the spin angular momentum (SAM) of double-index cylindrical vector beams in tight focus. Such a set of beams is a generalization of the conventional cylindrical vector beams since the polarization order is different for the different transverse field components. Based on the Richards-Wolf theory, we obtain an expression for the SAM distribution and show that if the polarization orders are of different parity, then the spin Hall effect occurs in the tight focus, which is there are alternating areas with positive and negative spin angular momentum, despite linear polarization of the initial field. We also analyze the orbital angular momentum spectrum of all the components of the focused light field and determine the overwhelming angular harmonics. Neglecting the weak harmonics, we predict the SAM distribution and demonstrate the ability to generate the focal distribution where the areas with the positive and negative spin angular momentum reside on a ring and are alternating in pairs, or separated in different semicircles. Application areas of the obtained results are designing micromachines with optically driven elements.

## 1. Introduction

In micromachines, elements can be driven by light [1,2]. This requires designing optical tweezers appropriate for driven elements, depending on their shape, material, and motion trajectory. The work [3] discusses how to control the mechanical motions of various particles in optical tweezers under complicated actuation of optical forces and torques by tightly focused laser beams.

Typically, a light beam comes out from a laser with a Gaussian shape. Then, for certain applications, not only for optical trapping, but also for optical data transmission and laser welding, the beam should be converted to attain an on-demand shape. For this purpose, a huge branch of modern optics, laser beam shaping, is developed [4].

The beam shaping techniques are developed both within a resonator and outside it [5], by using refractional or diffractional optical elements. External beam shaping can be done to shape a beam that maintains this shape on propagation [6], or in some specific area, for instance, in the focal plane [7].

However, in optical trapping, there can also be a need not only to trap a particle at a certain point, but also to make her do some movements. For instance, the particle can be forced to travel along some trajectory, or rotate around its own center. Such a rotation occurs when the light possesses the spin angular momentum (SAM) [8], or nonlinear polarization. Thus, in addition to the task of shaping the beam intensity distribution, there can be a task of shaping the SAM distribution. In addition to optical trapping, the SAM can be used as information in optical data transmission [9].

In paraxial approximation, the intensity shaping can be done for a single transverse field component of a homogeneously polarized light. However, for shaping the SAM, both transverse components should be tailored with a controlled phase delay between them. The problem becomes more difficult under tight focusing conditions.

Recently, it has been observed that when a linearly polarized light beam is tightly focused, then, near the focus, areas occur with elliptic polarization [10]. Since the areas with negative and positive SAM are spatially separated, this phenomenon is a manifestation of the optical spin Hall effect. Later, the same effect was discovered for tightly focused high-order cylindrical vector beams [11]. In [11], the SAM is distributed mostly on a ring and consists of alternating areas with positive and negative values. The cylindrical vector beams have the Jones vector **J** = [cos *m*φ, sin *m*φ] with φ being the angular polar coordinate and *m* being the polarization order (for *m* = 1, radial polarization). A further generalization is a two-index polarization singularity with the Jones vector **J** = [cos *m*φ, sin *n*φ], where *m* ≠ *n* [12], i.e., such a generalized vector field has different orders on the different axes. Recently, we studied such fields with V-points and for many values *m* and *n* we obtained the Poincare-Hopf index analytically [13].

In this paper, based on the Richards-Wolf theory [14], we study what happens with the SAM of a light field with the double-index polarization singularity in tight focus. We obtain an expression for the complex amplitude near the focus. Then, based on this expression, we derive the formula for the SAM and found that it can be nonzero only for the orders *m* and *n* of different parity. For analytical prediction of the SAM distribution, we decomposed the in-focus light field into the orbital angular momentum (OAM) spectrum and estimated the contribution of each angular harmonic. It turns out that if a light field being focused is not of ring shape and has a homogeneous or decaying Gaussian shape, then the OAM-spectrum consists mainly of *m*th and *n*th angular harmonics which exceed the other harmonics by an order of magnitude. It allows us to estimate the polar angles with zero SAM and thus to predict the SAM distribution. As an example, we obtained SAM distributions on a ring where the areas with positive and negative SAM occur in pairs.

## 2. A Light Field with a Double-Index Polarization Singularity near the Tight Focus

**Figure 1:**
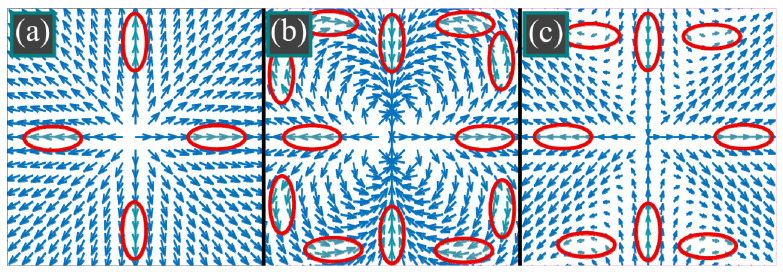
Conventional radial polarization (*m* = *n* = 1) (**a**), third-order radial polarization (*m* = *n* = 3) (**b**), double-index polarization singularity (*m* = 1, *n* = 3) (**c**). For radial polarization (**a**), there are two angles (0 and π) with a horizontal electric vector and two angles (π/2 and 3π/2) with a vertical electric vector. For 3rd-order radial polarization (**b**), there are six angles with horizontal electric vectors and six angles with vertical electric vectors. For the field with double-index polarization singularity of the order (1, 3), there are six angles with horizontal electric vectors and two angles with vertical electric vectors.

In [13], we investigated a generalization of cylindrical vector beams, when the polarization indices of the *E_x_* and *E_y_* field components were different. The amplitude of the electric vector of such a field is given by

(1) $$E\left(\theta ,\varphi \right)=A(\theta )\left(\begin{array}{l} \cos m\varphi \\ \sin n\varphi \end{array}\right),$$

where **E** is the strength vector of the electric field, *φ* is the azimuthal angle in the source plane, (*m*, *n*) is the two-index polarization order, θ is the polar angle, describing the tilt of the light rays to the optical axis, *A*(*θ*) is the amplitude of the source field as a function of the axis tilt angle. Directions of the electric vectors are illustrated in Figure 1.

In our work [15], we have obtained expressions for the Cartesian components of a linearly polarized optical vortex, focused on an aplanatic system. If an optical vortex with a topological charge *m* is linearly polarized along the axis *x*, then, in the input plane, the electric field is given by

(2) $$E\left(\theta ,\varphi \right)=A\left(\theta \right)\exp\left(im\varphi \right)\left(\begin{array}{c} 1\\ 0 \end{array}\right),$$

whereas near the tight focus, the complex amplitude reads as

(3) $$\left\{\begin{array}{l} E_{x}\left(\rho ,\psi ,z\right)=-\frac{1}{2}i^{m+1}e^{im\psi }\left(2I_{0,m}+e^{2i\psi }I_{2,m+2}+e^{-2i\psi }I_{2,m-2}\right),\\ E_{y}\left(\rho ,\psi ,z\right)=\frac{1}{2}i^{m}e^{im\psi }\left(e^{-2i\psi }I_{2,m-2}-e^{2i\psi }I_{2,m+2}\right),\\ E_{z}\left(\rho ,\psi ,z\right)=i^{m}e^{im\psi }\left(e^{-i\psi }I_{1,m-1}-e^{i\psi }I_{1,m+1}\right), \end{array}\right.$$

where (*ρ*, *ψ*, *z*) are the cylindrical coordinates with the origin in the focus.

In Equation (3), the functions *I_ν,μ_* are defined as follows:

(4) $$I_{\nu ,\mu }=2kf\int_{0}^{\alpha }\sin^{\nu +1}\left(\frac{\theta }{2}\right)\cos^{3-\nu }\left(\frac{\theta }{2}\right)\cos^{1/2}\left(\theta \right)A\left(\theta \right)e^{ikz\cos\theta }J_{\mu }\left(kr\sin\theta \right)d\theta ,$$

where *k* = 2π/λ is the wavenumber of monochromatic light with the wavelength λ, *f* is the focal length of the focusing lens, α is the maximal tilt angle of rays to the optical axis, defining the numerical aperture of the aplanatic lens NA = sin(α), *J_ν_* is the *ν*th-order Bessel function of the first kind.

The same way, if such an input field is linearly polarized along the axis *y*, the field components near the tight focus are equal to

(5) $$\left\{\begin{array}{l} E_{x}\left(\rho ,\psi ,z\right)=-\frac{1}{2}i^{m}e^{im\psi }\left(e^{2i\psi }I_{2,m+2}-e^{-2i\psi }I_{2,m-2}\right),\\ E_{y}\left(\rho ,\psi ,z\right)=-\frac{1}{2}i^{m+1}e^{im\psi }\left(2I_{0,m}-e^{2i\psi }I_{2,m+2}-e^{-2i\psi }I_{2,m-2}\right),\\ E_{z}\left(\rho ,\psi ,z\right)=i^{m+1}e^{im\psi }\left(e^{i\psi }I_{1,m+1}+e^{-i\psi }I_{1,m-1}\right). \end{array}\right.$$

The field with circularly symmetric amplitude distribution *A*(*θ*) and with polarization (1) can be decomposed into a superposition of four linearly polarized optical vortices:

(6) $$\begin{array}{l} E=A\left(\theta \right)\left(\begin{array}{l} \cos m\varphi \\ \sin n\varphi \end{array}\right)=\frac{1}{2}A\left(\theta \right)e^{im\varphi }\left(\begin{array}{c} 1\\ 0 \end{array}\right)+\frac{1}{2}A\left(\theta \right)e^{-im\varphi }\left(\begin{array}{c} 1\\ 0 \end{array}\right)\\ \;\;\;\;+\frac{-i}{2}A\left(\theta \right)e^{in\varphi }\left(\begin{array}{c} 0\\ 1 \end{array}\right)+\frac{i}{2}A\left(\theta \right)e^{-in\varphi }\left(\begin{array}{c} 0\\ 1 \end{array}\right). \end{array}$$

Using this decomposition, we get the field components of the field with polarization (1) near the tight focus:

(7) $$\begin{array}{c} E_{x}\left(\rho ,\psi ,z\right)=-\frac{1}{2}i^{m+1}\left\{2I_{0,m}\cos m\psi +I_{2,m+2}\cos\left[\left(m+2\right)\psi \right]+I_{2,m-2}\cos\left[\left(m-2\right)\psi \right]\right\}\\ \;+\frac{1}{2}i^{n+1}\left\{I_{2,n+2}\cos\left[\left(n+2\right)\psi \right]-I_{2,n-2}\cos\left[\left(n-2\right)\psi \right]\right\},\\ E_{y}\left(\rho ,\psi ,z\right)=-\frac{1}{2}i^{n+1}\left\{2I_{0,n}\sin n\psi -I_{2,n+2}\sin\left[\left(n+2\right)\psi \right]-I_{2,n-2}\sin\left[\left(n-2\right)\psi \right]\right\}\\ \;+\frac{1}{2}i^{m+1}\left\{I_{2,m-2}\sin\left[\left(m-2\right)\psi \right]-I_{2,m+2}\sin\left[\left(m+2\right)\psi \right]\right\}\\ E_{z}\left(\rho ,\psi ,z\right)=i^{m}\left\{I_{1,m-1}\cos\left[\left(m-1\right)\psi \right]-I_{1,m+1}\cos\left[\left(m+1\right)\psi \right]\right\}\\ \;+i^{n}\left\{I_{1,n+1}\cos\left[\left(n+1\right)\psi \right]+I_{1,n-1}\cos\left[\left(n-1\right)\psi \right]\right\}. \end{array}$$

The longitudinal component of the spin angular momentum is defined as [16]:

(8) Sz=2ImEx*Ey.

For simplicity, we suppose that the functions *A*(*θ*) are real valued. Thus, all the integrals *I_ν,μ_* are also real valued in the focal plane (*z* = 0). Then, substituting the transverse field components from Equation (7), we get the following expression:

(9) Sz=12Imin−m2I0,mcosmψ+I2,m+2cosm+2ψ+I2,m−2cosm−2ψ    ×2I0,nsinnψ−I2,n+2sinn+2ψ−I2,n−2sinn−2ψ    +12Imim−nI2,n+2cosn+2ψ−I2,n−2cosn−2ψ    ×I2,m−2sinm−2ψ−I2,m+2sinm+2ψ.

This expression is cumbersome, but it reveals that the SAM is zero unless the polarization orders *n* and *m* are of different parity. This is in contrast with [13], where we obtain the polarization singularity index of light fields with polarization from Equation (1). This index is, vice versa, nonzero only for the orders *n* and *m* of the same parity.

When the parity is the same for both orders, then polarization is linear in the focus, since, according to Equation (7), both *E_x_* and *E_y_* are proportional to *i^m^*^+1^ (or *i^n^*^+1^), multiplied by some real-valued function. Near the center (*r* << λ), if *n* > *m* ≥ 2, the transverse components *E_x_* and *E_y_* are approximately proportional to the vector **J** = [cos (*m*−2)φ, –sin (*m*−2)φ]. If *m* > *n* ≥ 2, they are proportional to the vector **J** = [cos (*n*−2)φ, –sin (*n*−2)φ]. Thus, a saddle-type polarization singularity is generated in the center [17].

For *n* and *m* of different parity, simplifications yield

(10) Sz=12Imin−m4I0,mI0,ncosmψsinnψ−2I0,mI2,n+2cosmψsinn+2ψ−2I0,mI2,n−2cosmψsinn−2ψ+2I0,nI2,m+2sinnψcosm+2ψ+2I0,nI2,m−2sinnψcosm−2ψ+I2,n+2I2,m+2+I2,n−2I2,m−2sinm−nψ−I2,m+2I2,n−2+I2,m−2I2,n+2sinm+nψ.

This equation is hard to analyze without knowing which terms contribute the most. Thus, we need to decompose the near-focus field into the angular harmonics and study its OAM-spectrum.

## 3. Balance of Light Field Energy near the Tight Focus

At first, we study the OAM-spectrum of linearly polarized light after tight focusing. According to Equation (3), if the input field is polarized along the axis *x*, then the *x*-component of the electric vector near the focus consists of three angular harmonics, whose topological charges are *m*, *m*−2, *m* + 2. The *y*-component consists of only two angular harmonics with the topological charges are *m*−2 and *m* + 2, whereas the *z*-component also consists of two angular harmonics with the topological charges are *m*−1 and *m* + 1. Each harmonic is proportional to the function *I_ν,μ_* from Equation (4). Therefore, such a harmonic has the following energy *W_ν,μ_* (see Appendix A):

(11) $$W_{\nu ,\mu }=4\pi f^{2}\int_{0}^{\alpha }\sin^{2\nu +1}\left(\frac{\theta }{2}\right)\cos^{5-2v}\left(\frac{\theta }{2}\right){\left|A\left(\theta \right)\right|}^{2}d\theta .$$

This expression indicates that the energy of the angular harmonic is independent of the distance *z* from the focal plane and of the topological charge of the optical vortex that determines the index *μ*.

The integrals (11) can be evaluated analytically only in simple cases, but, nevertheless, the contribution of each angular harmonic can be estimated. For example, if the field being focused is a uniform field with a constant amplitude *A*(*θ*) ≡ 1, then

(12) $$W_{0,\mu }=4\pi f^{2}\int_{0}^{\alpha }\sin\left(\frac{\theta }{2}\right)\cos^{5}\left(\frac{\theta }{2}\right)d\theta =\frac{4}{3}\pi f^{2}\left[1-\cos^{6}\left(\frac{\alpha }{2}\right)\right],$$

(13) $$W_{1,\mu }=4\pi f^{2}\int_{0}^{\alpha }\sin^{3}\left(\frac{\theta }{2}\right)\cos^{3}\left(\frac{\theta }{2}\right)d\theta =\frac{2}{3}\pi f^{2}\sin^{4}\left(\frac{\alpha }{2}\right)\left[1+2\cos^{2}\left(\frac{\alpha }{2}\right)\right],$$

(14) $$W_{2,\mu }=4\pi f^{2}\int_{0}^{\alpha }\sin^{5}\left(\frac{\theta }{2}\right)\cos\left(\frac{\theta }{2}\right)d\theta =\frac{4}{3}\pi f^{2}\sin^{6}\left(\frac{\alpha }{2}\right).$$

According to Equation (3), the *x*-component *E_x_* consists of three angular harmonics with their amplitude being proportional to the functions *I*_0,*m*_, *I*_2,*m*+2_/2, *I*_2,*m*–2_/2. The component *E_y_* is a superposition of harmonics described by the functions *I*_2,*m*+2_/2 and *I*_2,*m*–2_/2. Finally, the component *E_z_* is a superposition of harmonics described by the functions *I*_1,*m*+1_ and *I*_1,*m*–1_. Therefore, the total energy of the focal field is

(15) $$W=\left(W_{0,m}+\frac{1}{4}W_{2,m+2}+\frac{1}{4}W_{2,m-2}\right)+\left(\frac{1}{4}W_{2,m+2}+\frac{1}{4}W_{2,m-2}\right)+\left(W_{1,m+1}+W_{1,m-1}\right).$$

The brackets in Equation (15) illustrate, respectively, the energies of angular harmonics in the *E_x_*, *E_y_*, and *E_z_* field components. Since *W*_2,*m*+2_ = *W*_2,*m*–2_ and *W*_1,*m*+1_ = *W*_1,*m*–1_, we get

(16) $$W=W_{0,m}+W_{2,m+2}+2W_{1,m+1}.$$

Substitution of Equations (12)–(14) into Equation (16) yields

(17) $$W=\frac{4}{3}\pi f^{2}\left[1-\cos^{6}\left(\frac{\alpha }{2}\right)\right]\;+\frac{4}{3}\pi f^{2}\sin^{6}\left(\frac{\alpha }{2}\right)\;+\frac{4}{3}\pi f^{2}\sin^{4}\left(\frac{\alpha }{2}\right)\left[1+2\cos^{2}\left(\frac{\alpha }{2}\right)\right].$$

After simplifications, we get

(18) $$W=2\pi f^{2}\left(1\;-\cos\alpha \right).$$

This expression is exactly the size of a part of a sphere bound by the polar angle α. Thus, if a light field with unit amplitude is converging from a spherical surface with a numerical aperture sin(α), this field has exactly the energy given by Equation (18). This means that we found the balance when the energy of the input field is equal to the sum of energies of all angular harmonics of all three Cartesian components of the electric field in the focus.

**Figure 2:**
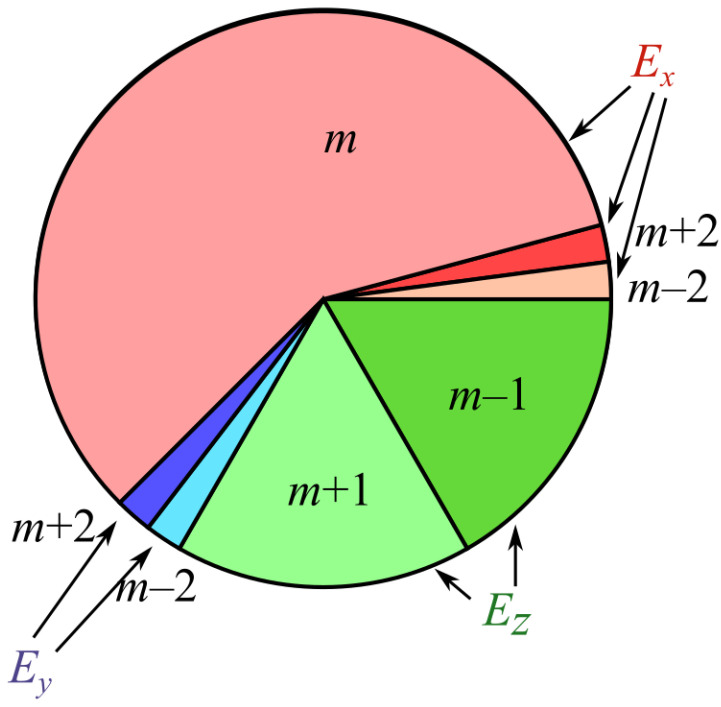
Energy distribution of a tightly focused linearly polarized optical vortex by the field components and by the angular harmonics.

In an extreme case, when the numerical aperture is close to the unit, i.e., α ≈ π/2, we get $W_{0,\mu }=(7/6)\pi f^{2}$, $W_{1,\mu }=(1/3)\pi f^{2}$, $W_{2,\mu }=(1/6)\pi f^{2}$. The whole energy is coinciding with the square of a hemisphere: *W* = *W*_0,*m*_ + *W*_2,*m*+2_ + 2*W*_1,*m*+1_ = 2π*f* ^2^. Thus, the total energy *W* of the input field is distributed in the focal field in the proportions shown in Figure 2. One third *W*/3 goes into the longitudinal component *E_z_*, and 2*W*/3 goes into the transverse components *E_x_* (5*W*/8) and *E_y_* (*W*/24). The energy of the component *E_x_* is distributed into the *m*th-order angular harmonic (7*W*/12) and into the harmonics of the orders *m* − 2 and *m* + 2, each of the energy *W*/48. The energy of the component *E_y_* is distributed equally in the angular harmonics of the orders *m* − 2 and *m* + 2, each of the energy *W*/48. The energy of the component *E_z_* is distributed equally in the angular harmonics of the orders *m* − 1 and *m* + 1, each of the energy *W*/6.

Obviously, if the input field is linearly polarized along the axis *y*, the energy distribution is the same, but the main portion (7*W*/12, or 58%) goes into the *m*th-order of the angular harmonic of the *y*-component *E_y_*.

The above energy proportions can change if the field intensity is not homogeneous, i.e., if *A*(*θ*) ≠ 1. However, if the amplitude function *A*(*θ*) decays from the center to the periphery, then the contribution of the *m*th-order angular harmonic becomes even greater. Indeed, for instance, if the aperture is bounded by an angle α, then the energy of the side angular harmonics of the orders *m* + 2 and *m*−2 relative to the energy of the central *m*th-order harmonic is

(19) $$\frac{W_{2,m}}{W_{0,m}}=\frac{\sin^{6}\left(\alpha /2\right)}{1-\cos^{6}\left(\alpha /2\right)}=\frac{\sin^{4}\left(\alpha /2\right)}{1+\cos^{2}\left(\alpha /2\right)+\cos^{4}\left(\alpha /2\right)}.$$

when α decreases from π/2 to 0, the numerator also decreases, while the denominator increases. Thus, this portion decays.

For instance, if α = π/2, then *W*_2,*m*_/*W*_0,*m*_ = 1/7 ≈ 0.143, but even if sin α = 0.95, then *W*_2,*m*_/*W*_0,*m*_ ≈ 0.057, i.e., almost all energy goes into the central *m*th-order harmonic.

**Figure 3:**
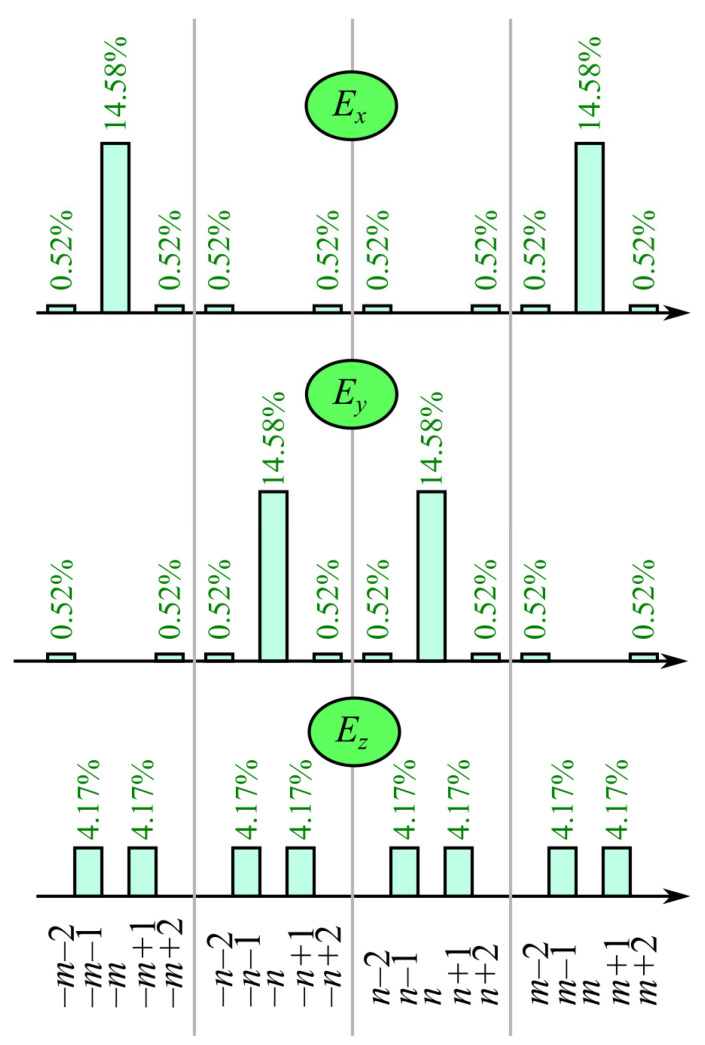
Energy distribution of a tightly focused light field with double-index polarization singularity by the field components and by the angular harmonics. Numbers above each pillar indicate the fraction of the whole energy of the focused light field that goes to the given angular harmonic of the given field component (the sum of all numbers is 100%).

Now we apply the above introduced technique for obtaining the OAM-spectrum of a light field with the double-index polarization singularity (1). Actually, the input field consists of four optical vortices of the orders *m*, –*m*, *n*, and –*n*. In the tight focus, each of these vortices splits into the several harmonics with the above derived energies. We suppose here for simplicity that α = π/2 and that these harmonics do not coincide with each other. Thus, we have the energy distribution (OAM-spectra of the components *E_x_*, *E_y_*, and *E_z_*), illustrated in Figure 3.

As seen in Figure 3, side angular harmonics of the orders *m* ± 2 and *n* ± 2 have relatively low energy (28 times lower than that of the orders *m* and *n*), and this energy becomes even lower when the aperture angle starts to decrease from α = π/2.

## 4. Spin Angular Momentum of Double-Index Polarization Vortices in a Tight Focus

Since it was found that almost all of the energy of the transverse field components goes into the *m*th-order and *n*th-order angular harmonics, we can suppose approximately that the SAM from Equation (10) reduces simply to

(20) Sz≈2Imin−mI0,mI0,ncosmψsinnψ.

Thus, it is seen that the SAM is equal to zero at the following polar angles:

(21) $$\begin{array}{l} \psi_{1,p}=\frac{\pi p}{n},\;p=0,\ldots ,2n-1,\\ \psi_{2,q}=\frac{\pi +2\pi q}{2m},\;q=0,\ldots ,2m-1. \end{array}$$

At certain conditions, these angles can coincide. For example, if *n* = 2*m*, we get

(22) Sz≈4ImimI0,mI0,2msinmψcos2mψ.

This indicates that there are 4*m* lines with zero SAM, starting from the origin and tilted with the polar angles

(23) $$\psi =\frac{\pi p}{2m}$$

with *p* = 0, 4*m* − 1.

However, at odd *p*, the cosine in Equation (22) is zero, but it is squared. This means that the SAM does not change its sign at these angles. Instead, due to the square, there is a second-order edge dislocation. At even *p*, the edge dislocation has the first order, and the SAM changes its sign. In comparison with the first-order dislocations, the dislocations of the second order looks like wider dark areas between the maxima. Thus, the SAM distribution should look like a set of pairs of the spots with positive and negative SAM.

Another case occurs when *m* = 1. For the SAM to be nonzero, *n* should be even. Thus, the angles α = ±π/2 are again the lines of second-order edge dislocation, where the SAM does not change sign. At other angles, the SAM changes the sign.

If *m* and *n* are relatively large and close to each other, the roots of the sine and cosine do not coincide but are close to each other. Therefore, the SAM changes its sign at each such angle, but, due to the pairs of close zeros, the SAM between them is insignificant.

## 5. Simulation

**Figure 4:**
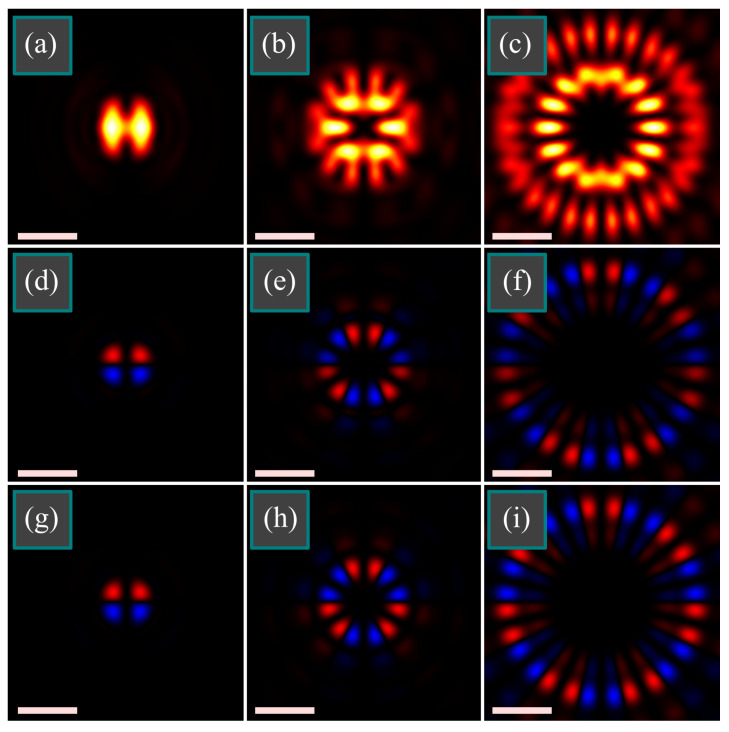
Distributions of intensity |*E_x_*|^2^ + |*E_y_*|^2^ + |*E_z_*|^2^ (**a**–**c**) and of longitudinal SAM density 2Im{E_x_*E_y_} (**d**–**i**) of tightly focused light field with double-index polarization singularity of the orders (*m*, *n*) = (1, 2) (**a**,**d**,**g**), (*m*, *n*) = (3, 6) (**b**,**e**,**h**), (*m*, *n*) = (7, 14) (**c**,**f**,**i**) at the following parameters: wavelength λ = 532 nm, focal length of the lens *f* = 10 μm, numerical aperture sin α = 0.95, amplitude apodization function is homogeneous, i.e., *A*(*θ*) ≡ 1. SAM distributions were computed directly by Equation (8) (**d**–**f**) and by an approximate Equation (20) (**g**–**i**). All the figures have the size 4 × 4 μm^2^ (scale mark shows 1 μm). Light and dark colors in the intensity distribution mean maximum and zero. Red and blue colors (**d**–**i**) mean, respectively, positive and negative SAM.

Numerical simulation was done by the Richards-Wolf equations. At first, we studied the case when *n* = 2*m*. Figure 4 illustrates the intensity |*E_x_*|^2^ + |*E_y_*|^2^ + |*E_z_*|^2^ and the longitudinal SAM density of a tightly focused light field with double-index polarization singularity of three different orders (*m*, *n*): (1, 2) (Figure 4a,d,g), (3, 6) (Figure 4b,e,h), (7, 14) (Figure 4c,f,i) at the following parameters: wavelength λ = 532 nm, focal length of the lens *f* = 10 μm, numerical aperture sin α = 0.95, amplitude apodization function is homogeneous, i.e., *A*(*θ*) ≡ 1. The SAM density distribution was computed directly by Equation (8) and then it was compared with the one computed by Equation (10). The distributions were visually the same, relative error was computed as max|*S_z_*_(10)_–*S_z_*_(8)_|/max|*S_z_*_(8)_| (where *S_z_*_(8)_ and *S_z_*_(10)_ are, respectively, SAM densities computed by Equation (8) and by Equation (10)). Maximal relative error was at (*m*, *n*) = (7, 14) and equal = 1.9 × 10^−15^. The third row of Figure 4 illustrates the approximate SAM distributions obtained by Equation (20). Formally, the relative error from the distributions obtained directly by Equation (8) is large (14–32%), but it is seen in Figure 4 that it almost does not affect the shape of the distribution. This is because the error was caused by neglecting the side angular harmonics of the order *m* ± 2 and *n* ± 2, and since, according to Figure 3, there are 16 such harmonics, each of them separately is weak and cannot reshape the SAM distribution significantly.

As seen in Figure 4, indeed, the SAM distribution consists of alternating pairs of spots with positive or negative SAM. This is different from the patterns we obtained earlier near the focus when the spots with positive and negative SAM were alternating in singles rather than in pairs [11].

**Figure 5:**
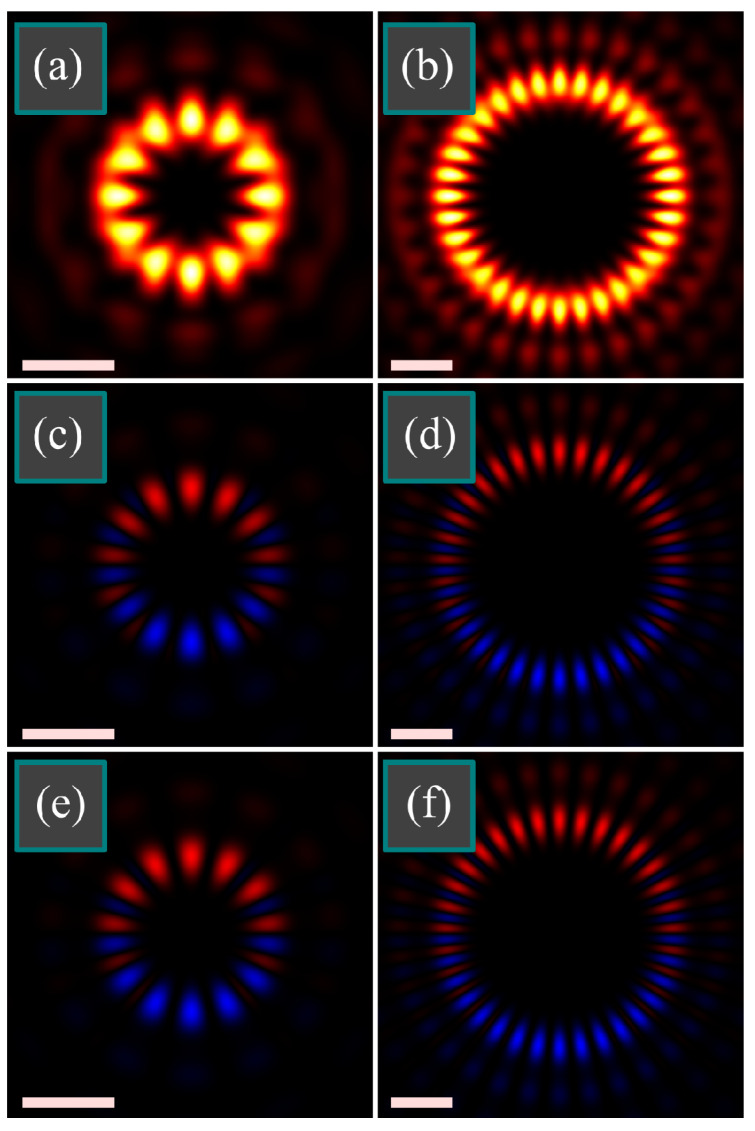
Distributions of intensity |*E_x_*|^2^ + |*E_y_*|^2^ + |*E_z_*|^2^ (**a**,**b**) and of longitudinal SAM density 2Im{E_x_^*^E_y_} (**c**–**f**) of tightly focused light field with double-index polarization singularity of the orders (*m*, *n*) = (6, 7) (**a**,**c**,**e**) and (*m*, *n*) = (16, 17) (**b**,**d**,**f**) at the following parameters: wavelength λ = 532 nm, focal length of the lens *f* = 10 μm, numerical aperture sin α = 0.95, amplitude apodization function is homogeneous, i.e., *A*(*θ*) ≡ 1. SAM distributions were computed directly by Equation (8) (**c**,**d**) and by an approximate Equation (20) (**e**,**f**). Figures have the size 4 × 4 μm^2^ (**a**,**c**,**e**) and 6 × 6 μm^2^ (**b**,**d**,**f**) (scale mark shows 1 μm). Light and dark colors in the intensity distribution mean maximum and zero. Red and blue colors (**c**–**f**) mean, respectively, positive and negative SAM.

Figure 5 illustrates the intensity and the longitudinal SAM density of tightly focused light field with double-index polarization singularity of two different orders (*m*, *n*): (6, 7) (Figure 5a,c,e) and (16, 17) (Figure 5b,d,f) at the following parameters: wavelength λ = 532 nm, focal length of the lens *f* = 10 μm, numerical aperture sin α = 0.95, amplitude apodization function is homogeneous, i.e., *A*(*θ*) ≡ 1. For comparison, the SAM distributions were also computed directly by Equation (8) and approximately by Equation (20). The relative error is again large (14% for both fields), but the shape of the SAM distribution is almost undistorted.

According to theoretical predictions, polar angles with zero SAM should occur by pairs of close angles. Figure 5 confirms it. It is seen that the positive SAM is mostly in the upper side while the negative SAM is mostly in the bottom side. Actually, the SAM is alternating, but between each spot with the positive or negative SAM, there is a weak spot of the opposite SAM, which is almost invisible in Figure 5.

**Figure 6:**
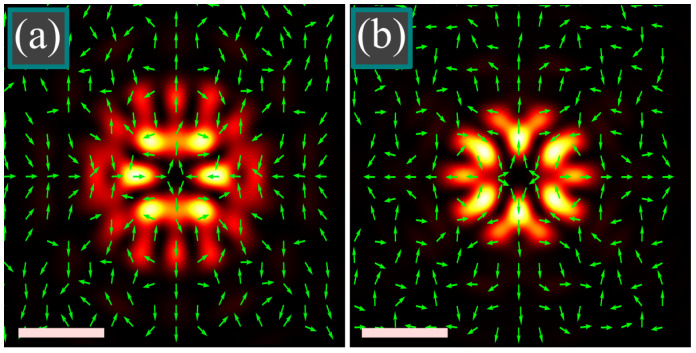
Intensity distribution and polarization directions of a tightly focused light field with double-index polarization singularity of the orders (*m*, *n*) = (3, 7) (**a**) and (*m*, *n*) = (5, 3) (**b**) at the following parameters: wavelength λ = 532 nm, focal length of the lens *f* = 10 μm, numerical aperture sin α = 0.95, radial apodization function is *A*(*θ*) ≡ 1. Scale mark shows 1 μm.

The above theory predicts that the SAM is zero for the orders *m* and *n* of the same parity. Computation confirms this and polarization of the focal field is thus linear. Figure 6 depicts the intensity distributions and the polarization directions of tightly focused light fields with double-index polarization singularity of the orders (*m*, *n*) = (3, 7) (Figure 6a) and (*m*, *n*) = (5, 3) (Figure 6b) with the rest parameters being the same as in Figure 4 and Figure 5: wavelength λ = 532 nm, focal length of the lens *f* = 10 μm, numerical aperture sin α = 0.95, radial apodization function *A*(*θ*) ≡ 1.

It is seen in Figure 6 that *E_y_* = 0 on the horizontal axis (φ = 0 and φ = π) and *E_x_* = 0 on the vertical axis (φ = ±π/2), which is consistent with Equation (7) for the complex amplitudes of the light field. It is also seen that in both cases a saddle-type polarization [17] singularity is generated in the center.

## 6. Conclusions

Based on the Richards-Wolf theory, we have investigated here the spin angular momentum of double-index cylindrical vector beams in tight focus. Such a set of beams is a generalization of the conventional cylindrical vector beams since the polarization order is different for the different transverse field components. Thus, in the beam periphery, the number of areas with horizontal polarization is not equal to the number of areas with vertical polarization.

It turns out that if the polarization orders are of different parity, then the spin Hall effect occurs in the tight focus, which is there are alternating areas with positive and negative spin angular momentum, despite linear polarization of the initial light field.

On the contrary, if the polarization orders are of same parity, then polarization in the tight focus remains linear (but inhomogeneous).

For analytical description of the spin angular momentum distribution, we also analyzed the orbital angular momentum (OAM) spectrum of a linearly polarized *m*th-order vortex field in tight focus. It turns out that if the initial light field is not of ring shape and has a homogeneous or decaying Gaussian shape, then the energy of the angular harmonics with the orders *m* ± 2 in the transverse field components are at least 28 times lower than the energy of the *m*th-order angular harmonic.

This decomposition of the focused field into the OAM spectrum allowed us to predict the spin angular momentum distribution and, as an example, we demonstrated the ability to generate the focal distribution where the areas with the positive and negative spin angular momentum reside on a ring and are alternating in pairs or separated in different semicircles.

We limited our considerations by a homogeneous initial light field with constant amplitude and zero phase. Only polarization was supposed to be inhomogeneous. As a future work, light fields can be studied with the double-index polarization singularity, but with an inhomogeneous amplitude or/and phase distribution, like, for instance, circular Airy beams with vortices [18,19,20] or quadratic-power-exponent-phase vortex beams [21,22]. These beams were studied in homogeneous medium [18], uniaxial crystals [19,21], or in tight focusing conditions [20,22], but their tight focusing with double-index polarization singularities and the possibility of the optical spin Hall effect in the tight focus were not yet investigated.

Application areas of the results obtained are designing micromachines for optical driving biological objects [23,24] or microtools in a lab-on-a-chip [25]. In contrast to the orbital angular momentum, which causes microscopic particles to rotate along a ring, the spin angular momentum causes articles to rotate around their centers of mass [8] and tailoring the SAM density distribution can allow simultaneous manipulation by an ensemble of particles. For this purpose, the particles should be non-metallic, but absorbing, since the intensity maximums attract dielectric particles. The particles should be of a size comparable with the areas of positive or negative SAM density, e.g., 0.2 μm in the distributions from Figure 4. Such sizes can have, for instance, polystyrene beads [26]. Another application is optical information transmission where the SAM density distribution can be used for encoding the data.

## Data Availability

Not applicable.

## Appendix A. Derivation of the Energy of a Single Angular Harmonic Near the Tight Focus

As seen from the above expressions for the field near the tight focus, each angular harmonic is proportional to the function *I_ν,μ_* from Equation (4). Below we derive the energy *W_ν,μ_* of such a separate angular harmonic:

(A1)
$$W_{\nu ,\mu }=\int_{0}^{\infty }\int_{0}^{2\pi }{\left|I_{\nu ,\mu }\left(r,z\right)\right|}^{2}rdrd\varphi =2\pi \int_{0}^{\infty }{\left|I_{\nu ,\mu }\left(r,z\right)\right|}^{2}rdr.$$

Substituting here the function *I_ν,μ_* from Equation (4), we get

(A2)
$$\begin{array}{l} W_{\nu ,\mu }=8\pi k^{2}f^{2}\int_{0}^{\infty }\left[\int_{0}^{\alpha }\sin^{\nu +1}\left(\frac{\theta }{2}\right)\cos^{3-\nu }\left(\frac{\theta }{2}\right)\cos^{1/2}\left(\theta \right)A^{*}\left(\theta \right)e^{-ikz\cos\theta }J_{\mu }\left(kr\sin\theta \right)d\theta \right]\\ \;\;\;\;\times \left[\int_{0}^{\alpha }\sin^{\nu +1}\left(\frac{\theta^{\prime }}{2}\right)\cos^{3-\nu }\left(\frac{\theta^{\prime }}{2}\right)\cos^{1/2}\left(\theta^{\prime }\right)A\left(\theta^{\prime }\right)e^{ikz\cos\theta^{\prime }}J_{\mu }\left(kr\sin\theta^{\prime }\right)d\theta^{\prime }\right]rdr. \end{array}$$

Changing the integration variables *η* = sin *θ*, *η*′ = sin *θ*′ and changing the integration order, we obtain

(A3) $$\begin{array}{c} W_{v,\mu }=8\pi k^{2}f^{2}\int_{0}^{\sin\alpha }{\left(\frac{1-\sqrt{1-\eta^{2}}}{2}\right)}^{\frac{v+1}{2}}{\left(\frac{1+\sqrt{1-\eta^{2}}}{2}\right)}^{\frac{3-\nu }{2}}\\ \;\times {\left(1-\eta^{2}\right)}^{1/4}A^{*}\left(\arcsin\eta \right)\exp\left(-ikz\sqrt{1-\eta^{2}}\right)\frac{d\eta }{\sqrt{1-\eta^{2}}}\\ \;\times \int_{0}^{\sin\alpha }{\left(\frac{1-\sqrt{1-\eta^{\prime 2}}}{2}\right)}^{\frac{v+1}{2}}{\left(\frac{1+\sqrt{1-\eta^{\prime 2}}}{2}\right)}^{\frac{3-v}{2}}\\ \;\times {\left(1-\eta^{\prime 2}\right)}^{1/4}A\left(\arcsin\eta^{\prime }\right)\exp\left(ikz\sqrt{1-\eta^{\prime 2}}\right)\frac{d\eta^{\prime }}{\sqrt{1-\eta^{\prime 2}}}\\ \;\times \left[\int_{0}^{\infty }J_{\mu }\left(kr\eta \right)J_{\mu }\left(kr\eta^{\prime }\right)rdr\right]. \end{array}$$

Due to the orthogonality of the Bessel functions [27], the inner integral over *r* reduces to the Dirac delta function (if *μ* ≥ –1):

(A4)
$$\int_{0}^{\infty }J_{\mu }\left(k\eta r\right)J_{\mu }\left(k\eta^{\prime }r\right)rdr=\frac{\delta \left(k\eta -k\eta^{\prime }\right)}{k\eta }=\frac{1}{k^{2}}\frac{\delta \left(\eta -\eta^{\prime }\right)}{\eta },$$

Thus, Equation (A3) is simplified and only one integral remains:

(A5)
$$W_{\nu ,\mu }=8\pi f^{2}\int_{0}^{\sin\alpha }{\left(\frac{1-\sqrt{1-\eta^{2}}}{2}\right)}^{\nu +1}{\left(\frac{1+\sqrt{1-\eta^{2}}}{2}\right)}^{3-\nu }{\left|A\left(\arcsin\eta \right)\right|}^{2}\frac{d\eta }{\eta \sqrt{1-\eta^{2}}}.$$

Now we return back to the trigonometric functions *η* = sin *θ* and get

(A6)
$$\begin{array}{l} W_{\nu ,\mu }=8\pi f^{2}\int_{0}^{\alpha }{\left(\frac{1-\cos\theta }{2}\right)}^{\nu +1}{\left(\frac{1+\cos\theta }{2}\right)}^{3-\nu }{\left|A\left(\theta \right)\right|}^{2}\frac{d\theta }{\sin\theta }\\ \;\;\;\;=4\pi f^{2}\int_{0}^{\alpha }\sin^{2\nu +1}\left(\frac{\theta }{2}\right)\cos^{5-2v}\left(\frac{\theta }{2}\right){\left|A\left(\theta \right)\right|}^{2}d\theta . \end{array}$$
